# Newly developed genomic SSR markers revealed the population structure and genetic characteristics of abaca (*Musa textilis* Nee)

**DOI:** 10.5114/bta.2024.145255

**Published:** 2024-12-19

**Authors:** Mariecris Rizalyn R. Mendoza, Antonio C. Laurena, Maria Genaleen Q. Diaz, Eureka Teresa M. Ocampo, Tonette P. Laude, Antonio G. Lalusin

**Affiliations:** 1 Institute of Plant Breeding, College of Agriculture and Food Science, University of the Philippines Los Baños, Philippines; 2 Institute of Crop Science, College of Agriculture and Food Science, University of the Philippines Los Baños, Philippines; 3 Institute of Biological Sciences, College of Arts and Sciences, University of the Philippines Los Baños, Philippines

**Keywords:** abaca, AMOVA, DARwin, genetic diversity, genomic SSR markers, population structure

## Abstract

Abaca (*Musa textilis* Nee) is the primary source of manila hemp fiber, a vital industrial product for the country. Previous studies have relied on molecular markers designed for other *Musa* species or distant genera like rice, limiting accurate genetic characterization and germplasm conservation. To address this, we developed 50 genome-specific molecular markers based on the recently released whole genome sequence assembly of Abaca var. *Abuab* by Galvez et al. (2021). Among these markers, 28 showed high polymorphism, with an average PIC value of 0.78. Population analysis revealed a heterozygosity of 0.428, indicating moderate genetic diversity, supported by an alpha value of 0.0735 and an *F*_stp_ value of 0.0815, which suggests moderate genetic differentiation among abaca accessions. Cluster analyses, generated by DARwin and STRUCTURE software with 91% similarity, identified four clusters. The new markers were also able to distinguish six *Musa* accessions exhibiting morphological traits of both abaca and banana. Discrepancies in sample identification due to identical or inverted names were resolved using population structure analysis. Molecular variance analysis showed a 12% variance among the four abaca subpopulations and 88% within populations, suggesting recent divergence. Our study highlights the diversity, identity, and genetic variation within the abaca collection using accurate, robust, cost-effective, and computationally simple genome-specific markers. These markers are pivotal for genetic studies of abaca, including traitmarker mapping and the differentiation of accessions even in the juvenile stage, when phenotypic differences may be subtle.

## Introduction

The role of diversity analysis extends beyond the conservation and management of genetic resources (Salgotra and Chauhan, 2023); it is also critical for the continuity and success of plant breeding programs, which depend on access to a diverse population (Ebert et al., 2023). Molecular markers are widely used tools in diversity assessment, with simple sequence repeats (SSRs) or microsatellites and SNP markers being particularly valuable due to their ability to detect high genetic variability and their abundance in the genome (Zhu et al., 2016). Although SSRs are cost-effective, highly polymorphic markers, they are gradually replaced by SNP markers. However, breeding institutes with limited funds often prefer SSR markers due to their high polymorphism, computational simplicity, and accessibility to many molecular laboratories (Choudhury et al., 2023). The robustness of both marker systems has been demonstrated in crops such as rice (Singh et al., 2013), juniper (García et al., 2018), perennial ryegrass (Liu et al., 2018), and melon (Zhang et al., 2023). Each marker type has shown strengths in various diversity analysis aspects, such as SNPs for assessing geographic isolation and SSRs for parental assignment. Combining these marker technologies is useful for variety verification and DNA fingerprinting of large crop families like melon (Zhang et al., 2023).

Currently, there are eight *Musa* genome sequences available. Due to the ploidy level of *Musa* spp., Martin et al. (2016) used the double haploid Pahang to improve the assembly of the *M. acuminata* genome, which was chosen because it is one of the progenitors of domesticated Cavendish bananas. *M. acuminata* became the first *Musa* species and the first monocot outside the Poales order to have a draft genome sequence, establishing it as the model species for sequencing other *Musa* spp., such as *M. itenerans* (Wu et al., 2016), *M. balbisiana* Colla (Niu et al., 2018), and *M. banksii* (Liu et al., 2021). Studies on genomes of wild relatives like *M. itenerans* have provided insights into whole-genome duplication events that contributed to species divergence (Wu et al., 2016). Disease resistance elements identified in the genome sequences of these species also suggest the potential for gene introgression from wild relatives with both resistance and desirable agronomic traits, facilitating the development of *Musa* varieties that are well-adapted to multiple environments.

Abaca (*Musa textilis* Nee), an endemic fiber species from the Philippines, is clonally propagated and perennial. Approximately 84% of abaca fiber production is attributed to the Philippines (Halos, 2008). This natural fiber crop occupies unique market niches, such as paper bill manufacturing and the natural textile industry, highlighting the need to improve this high-value commodity to meet local and international demands. While vegetative propagation ensures that true-to-type traits are preserved across generations, it also results in a narrow genetic base and genomic erosion due to the lack of recombination (Liu et al., 2023). The distribution of this crop, along with its physical similarity to other *Musa* species, has led to misidentification and mismanagement within the abaca gene bank. Many abaca varieties have been given different names despite identical accessions (Halos, 2008).

The utility of molecular markers in breeding programs depends on marker quantity and quality (Kaldate et al., 2017). Most SSR markers used for abaca are banana-based or cross-transferred from non-*Musa* species. Of these, six banana-based markers have shown polymorphism within the abaca germplasm collection (Boguero et al., 2016; Yllano et al., 2020). Genome-wide SSR markers derived from publicly available whole genome assemblies have enhanced diversity analysis, linkage mapping, and marker – trait associations in several crops, including banana (Biswas et al., 2020), walnut (Itoo et al., 2023), grain amaranth (Vats et al., 2023), wheat (Fandade et al., 2023), and citrus (Singh et al., 2023). For instance, molecular markers developed from the genome assembly of flax have differentiated fiber and linseed types (Pan et al., 2020). Multiple studies have investigated critical components of abaca genomics and transcriptomics to elucidate the genetic composition of the crop. These include SSRs linked to economically significant diseases and fiber quality (Lalusin, 2010; Damo, 2011; Dizon et al., 2012; Pabro, 2012; Palao, 2018; Yllano et al., 2020), filtered genome analysis (Vilela et al., 2015), and transcriptome studies (Muncada, 2018; Reamillo, 2018). The release of the 616 Mbp abaca var. Abuab de novo whole genome assembly offers a valuable resource of molecular markers (Galvez et al., 2021). Genome-wide SSRs from the abaca genome assembly provide an efficient marker system to analyze genetic relatedness within the abaca gene bank, determine population structure, and facilitate varietal identification.

In this study, we developed, identified, and characterized 50 microsatellite markers to comprehensively assess the genetic diversity and variety identity of the UPLB (University of the Philippines Los Baños) abaca collection from 10 regions in the country (Supplementary Table 1). Using multiple genetic diversity software programs, we assessed the genetic relatedness of abaca and evaluated the markers’ efficiency in detecting other *Musa* species with physical similarities to abaca. Finally, we analyzed the collinearity of the results from these new markers across different software to evaluate their consistency in discrimination power.

## Materials and methods

### Genome SSR marker design

Before using the GMATA (Genome-wide Microsatellite Analyzing Tool Package; Wang and Wang, 2016) program to generate genome-wide SSR markers, specific preparatory steps were undertaken. In the phylogenetic tree established by Galvez et al. (2021), abaca (*M. textilis* Nee) was found to be more closely related to *M. balbisiana* Colla than to *M. acuminata*. Therefore, the *M. balbisiana* genome was used as the reference to create a reference-based chromosome assembly for *M. textilis* Nee.

The Chromosomer software (Tamazian et al., 2016) was employed to build a straightforward chromosome assembly based on the pairwise alignment of scaffolds and contigs to the reference genome. Fragments were aligned twice — once in the forward direction and once in reverse — against the reference. The *M. textilis* Nee genome, available at https://datadryad.org/stash/share/Yk6Ls1qw7WQts4zl03iPEchuiw6kMKBJBy6Oa1-JN00, was obtained from the Data Dryad repository (Galvez et al., 2021). The *M. balbisiana* Colla chromosome assembly was obtained from https://www.ncbi.nlm.nih.gov/datasets/genome/GCA_004837865.1/ (Wang et al., 2019). Only the mapped fragments of the *M. textilis* Nee genome were processed through the GMATA program.

To generate primer pairs, the mapped *M. textilis* Nee fragments were analyzed using GMATA under default settings: dinucleotide (2) to hexanucleotide (6) motif repeats, a minimum amplicon size of 120 bp, an optimal annealing temperature of 60°C, a minimum GC content of 40%, and a primer length of 18–25 bp. The SSR selection process described by Bhattarai et al. (2021) for spinach g-SSRs was then applied to filter the microsatellite markers. The following criteria were used to exclude markers from the study: 1) motifs without designed flanking regions, 2) markers with an AT/TA motif, and 3) loci within 100 bp of each other. Finally, markers showing polymorphism, as determined by the GMATA e-mapping program, were retained as the final set of SSR markers for this study.

### Plant materials and polymerase chain reaction

A total of 99 *Musa textilis* accessions, collected from 10 administrative regions of the Philippines, were used in this study (Supplementary Table 1). Six accessions from this collection exhibited morphological similarities to bananas. To confirm this observation, two dendrograms were generated (Fig. 3 and Fig. 10).

DNA was isolated using the Doyle and Doyle (1990) CTAB DNA extraction protocol, as modified by Sandoval et al. (2011). The PCR conditions were adapted from the protocols described by Boguero et al. (2016) and Yllano et al. (2020). Each 10 μl PCR reaction consisted of 1× PCR buffer, 2 mM MgCl_2_, 0.2 mM dNTPs, primers (forward and reverse), and 0.05 U/μl Taq polymerase cocktail. The PCR cycling conditions were as follows: initial denaturation at 94°C for 4 min; 35 cycles of denaturation at 94°C for 30 s, annealing at 58–59.9°C for 30 s, and extension at 72°C for 45 s; followed by a final extension at 72°C for 10 min. The amplified bands were visualized on a 6% polyacrylamide gel stained with GelRed and analyzed using the GenoSens photodocumentation system. Polymorphic bands were scored with the GelAnalyzer v.23.1 software (Lazar and Lazar, 2023).

### Data analysis

To comprehensively assess the genetic relatedness of the abaca collection, allele frequency, genetic distance, principal coordinate analysis (PCoA), and analysis of molecular variance (AMOVA) were determined using GenAIEx v.6.5 (Genetic Analysis in Excel) software (Peakall and Smouse, 2012). Following the Hardy–Weinberg Equilibrium (HWE) framework in GenAIEx, the expected heterozygosity was calculated using the formula:


$$H=1-\Sigma p_{i}^{2}$$

where *H* – diversity index, $\Sigma p_{i}^{2}$ – sum of squares of individual observation in the *i* ^th^ category.

The Jaccard index was computed in the Dissimilarity Analysis Representation for Windows (DARwin) software ver. 6.0.21 (Perrier and Jacquemoud-Collet, 2006) using the formula:


$$dij=\frac{b+c}{a+b+c}$$

where *dij* is the distance index between *i* and *j* ; *a* is the number of variables where *x_i_* – present and *x_j_* – present; *b* is the number of variables where *x_i_* – present and *x_j_* – absent; *c* is the number of variables where *x_i_* – absent and *x_j_*– present.

The unweighted neighbor-joining tree was constructed using DARwin software v.6.0.021, with 1,000 bootstrap repetitions. Population assignment for each accession was computed using STRUCTURE software v.2.3.4 (Pritchard et al., 2000). In this admixture model, a total of ten replications for each *K* value were performed, with each *K* run over a burn-in period of 20,000 iterations and 50,000 Monte Carlo Markov Chain (MCMC) replicates.

The optimal *K*-means cluster was determined using the R package Pophelper v.2.3.1 (Francis, 2017; https://github.com/royfrancis/pophelper). The degree of population differentiation among the four subpopulations was assessed using Hierfstat. To test the collinearity between the two clustering methods, a Venn diagram was generated using the Venny 2.1 analysis program (https://bioinfogp.cnb.csic.es/tools/venny/), developed by Oliveros (2007).

## Results

### Genome-wide SSR marker analysis

Since the only available genomic data for abaca consisted of scaffolds, the Chromosomer software was utilized to ensure the SSR markers were developed within the aligned scaffolds for greater accuracy. Chromosomer generated a chromosome assembly based on the alignment of the *M. textilis* Nee genome (query) with the *M. balbisiana* Colla genome (reference).

The Chromosomer software mapped the Abuab genome (Galvez et al., 2021) to the 11 chromosomes of *M. balbisiana* Colla, aligning 11,720 fragments or 221.5 Mbp of the 473 Mbp (-46.8%) *M. balbisiana* Colla chromosome assembly. This mapping produced 7,615 unlocalized fragments and 26,738 unplaced scaffolds. Only the mapped fragments were used to avoid developing markers from unlocalized or unplaced regions. A total of 50 genome-wide SSR markers were designed and applied to assess the diversity of the abaca germplasm collection. Among these, 28 polymorphic primers were identified and used for all subsequent analyses.

Since six abaca accessions exhibited morphological similarities with bananas, two clusters were generated to highlight the genetic and morphological differences between abaca and banana-like accessions. This was necessary, as natural hybrids such as Canton and Minay exist (Halos, 2008). The morphological similarities between abaca cultivars have historically resulted in misidentification, with many accessions sharing identical or similar names due to traditional propagation via suckers, which farmers often label with the same identification.

**Fig. 1:**
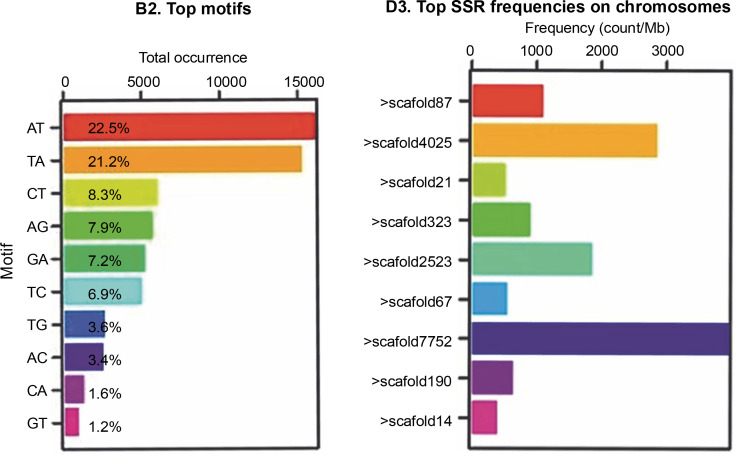
(A) SSR motif distribution and (B) frequencies of SSR markers distributed on the scaffolds of the molecular markers developed from the aligned Abuab genome (*Musa textilis* Nee) using the GMATA program

Figure 1 shows that the most abundant motifs in the Abuab genome assembly were AT and TA, representing 22.5 and 21.2%, respectively. However, these motifs were excluded due to scoring difficulties and reproducibility issues (Bhattarai et al., 2021). While the highest number of SSRs was found in > scaffold7752, none of the designed markers from this scaffold were polymorphic and therefore were not included in the study.

Table 1 details the 28 polymorphic SSR markers developed using the GMATA program, including motifs, fragment locations, corresponding *M. balbisiana* Colla chromosomes, annealing temperatures, expected product sizes, and PIC values. Chromosomes 2 and 3 were not represented. PIC values ranged from 0.49 to 0.91, with an average of 0.78, indicating that this marker set is highly informative and suitable for genetic analysis and variety fingerprinting.

**Table 1:** The profile of the polymorphic genome wide SSR markers used in assessing the diversity of the abaca (*Musa textilis* Nee) germplasm collection

MarkerID	Motif	SequenceID	*M. balbisiana* chromosome ID	Annealing temperature	Product size	PIC value
>MK8575	(CT)5	>scaffold800|8981:9790|9381:9390	1	59.99	334	0.81
>MK13267	(CT)8	>scaffold1488|13208:14023|13608:13623	1	60	172	0.81
>MK37483	(CT)8	>scaffold17668|4288:5103|4688:4703	1	60	333	0.88
>MK34686	(GA)6	>scaffold8297|1925:2736|2325:2336	4	59.5	386	0.88
>MK41914	(AC)5	>scaffold27280|1863:2672|2263:2272	4	60.3	248	0.67
>MK14907	(GA)14	>scaffold1813|52002:52829|52402:52429	5	60.1	352	0.88
>MK34525	(GA)	>scaffold8197|6057:6870|6457:6470	5	59.9	196	0.84
>MK39951	(TG)5	>scaffold16552|5399:6208|5799:5808	5	59.8	250	0.82
>MK41848	(AC)5	>scaffold17954|924:1733|1324:1333	5	60.1	174	0.72
>MK42523	(TG)5	>scaffold21334|1451:2260|1851:1860	5	60.1	174	0.49
>MK29084	(AC)6	>scaffold5595|2658:3469|3058:3069	5	59.4	350	0.82
>MK25159	(TC)9	>scaffold4137|45607:46048|46007:46024	6	59.9	293	0.84
>MK3225	(AG)5	>scaffold237|97822:98631|98222:98231	6	59.9	188	0.62
>MK34491	(TG)5	>scaffold8184|20743:21552|21143:21152	7	60.1	330	0.73
>MK2915	(GA)10	>scaffold214|20099:20918|20499:20518	8	60.31	360	0.91
>MK14233	(AG)12	>scaffold2286|65101:65824|65501:65524	8	59.94	286	0.78
>MK36557	(AC)5	>scaffold26065|292:1101|692:701	8	59.9	321	0.80
>MK34940	(AC)5	>scaffold8425|17891:18700|18291:18300	9	60.2	169	0.85
>MK12476	(GA)7	>scaffold1357|31206:32019|31606:31619	9	59.9	306	0.67
>MK20812	(TG)11	>scaffold2956|33342:34163|33742:33763	10	59.6	296	0.79
>MK12569	(AG)5	>scaffold1375|21641:22450|22041:22050	10	60.2	338	0.87
>MK36595	(TG)8	>scaffold33801|1:1885|335:350	10	60.1	384	0.53
>MK39269	(TG)	>scaffold12187|1:551|138:151	10	60.1	388	0.88
>MK44421	(TG)6	>scaffold40160|1:1404|84:95	10	60	209	0.74
>MK32415	(GA)5	>scaffold7071|24065:24874|24465:24474	11	60	285	0.77
>MK41610	(AC)5	>scaffold17165|933:1742|1333:1342	11	59.6	365	0.90
>MK43779	(GT)5	>scaffold31585|1366:2114|1766:1775	11	59.6	362	0.73

**Fig. 2:**
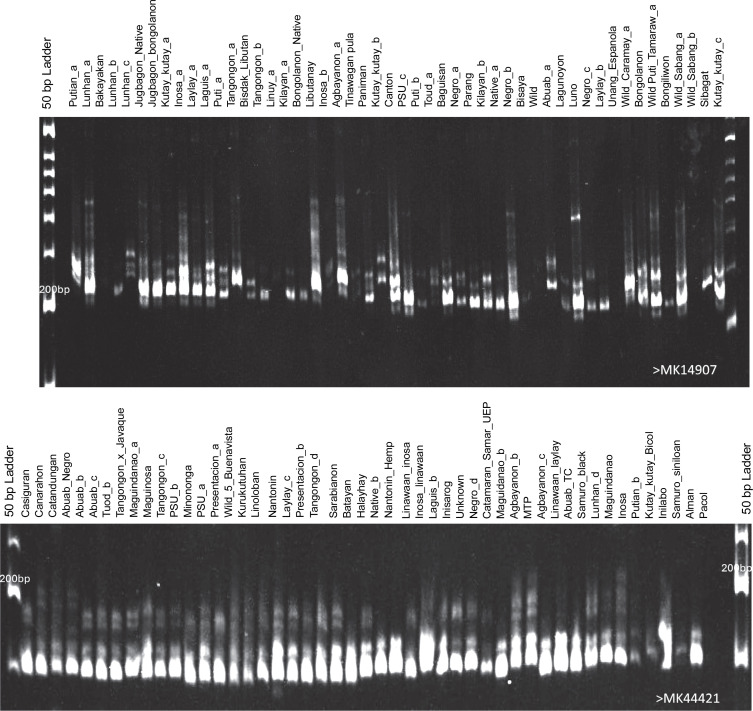
Representative 6% polyacrylamide gels showing the generated polymorphic bands of the abaca (*Musa textilis* Nee) accessions using markers MK14907 and MK44421

The average heterozygosity obtained from the 99 samples was 0.428 based on the HWE analysis of GENAIEx v.6.5 software, suggesting moderate heterogeneity. The population used in this study served as a germplasm collection with representative accessions from the ten administrative regions of the Philippines. Modified PCR protocols previously used in abaca molecular studies (Boguero et al., 2016; Yllano et al., 2020) were applied to the newly developed markers, resulting in bands ranging from 187 to 236 per population, with band frequencies $5% ranging from 173 to 193 (Fig. 2). Unique bands in each of the four subpopulations ranged from 16 to 48, while shared bands across # 50% of populations ranged from 33 to 50 alleles (Table 2). These unique bands may serve as distinct markers for specific abaca populations or accessions in future studies.

**Table 2:** Allelic distribution and band frequencies across the four abaca (*Musa textilis* Nee) subpopulations

Population	Pop1	Pop2	Pop3	Pop4
No. bands	236	230	187	246
No. bands Freq. ≥ 5%	173	179	187	194
No. private bands	48	32	16	42
No. LComm bands (≤ 50%)	44	46	33	50

The SSR markers developed from the *M. textilis* var. Abuab genome improved the specificity and accuracy of abaca genetic characterization. This research advances previous molecular studies of abaca, which relied on markers derived from the genomes of other *Musa* species or distant plant genera.

In this study, the SSR markers proved robust for diversity assessment, effectively delineating abaca accessions at the genetic level and confirming moderate diversity within the population. The SSR marker system offers rapid, accessible results and does not require a skilled bioinformatician or advanced computational resources, making it feasible for use in established molecular laboratories. The SNP marker system, on the other hand, is largely outsourced in the Philippines, and only a few agencies are capable of sequencing (i.e., Philippine Genome Centers); therefore, the SSR marker system is a good choice for studies dealing with genetic diversity.

### Cluster analysis of the 93 abaca (Musa textilis Nee) accessions

**Fig. 3:**
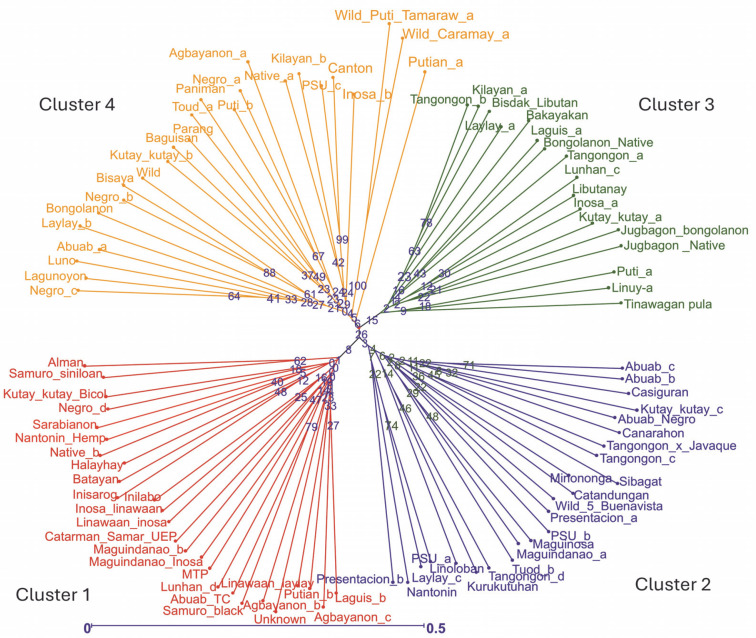
Diversity analysis of the 93 abaca accessions (*Musa textilis* Nee) using 28 polymorphic genome-wide microsatellite markers generated through the DARwin v.6.0 software

Two neighbor-joining trees were generated in this study. The first tree included only 93 abaca accessions (Fig. 3), while the second incorporated six accessions with morphological similarities to bananas to illustrate the genetic differences between these accessions and true abaca. The radial tree grouped the 93 abaca accessions into four clusters using an average Jaccard similarity index of 0.69, indicating moderate dissimilarity among accessions.

Cluster 1 consisted of 26 abaca accessions from nine administrative regions of the Philippines. Region 5 had the highest representation at 26.9%, followed by Regions 6, 8, and 13, each contributing approximately 15%. Notably, Region 5 is among the largest producers of abaca. Accessions Linawaan_inosa and Inosa_linawaan are grouped closely within this cluster. These accessions also showed high percent similarity among the subpopulations generated by population structure. The only Negro accession placed in cluster 1 was Negro_d, indicating its genetic difference from the other Negro accessions. This observation was confirmed in the population structure analysis. Both Samuro_black and Samuro_siniloan, which came from different areas (Region V and Region IV-A, respectively), but bear the Samuro name were found in this cluster. The Samuro cultivar is typically found in Region V (Halos, 2008). This region lies around 300km away from Laguna (Region IV-A) where Samuro_siniloan was found. Despite coming from the same region, only Agbayanon_a was separated from other accessions bearing the same label, suggesting that this cultivar might have originated from other regions.

Cluster 2 was composed of 24 abaca accessions that were collected from six regions. The Bicol region has the highest share in this group at 39%, followed by regions 4 and 12, which contributed 26 and 17%, respectively. The most notable result in this cluster was the Abuab cultivars. The Abuab_b, Abuab_c, and Abuab_negro collected from Region 5 were grouped in Cluster 2, while Abuab_a acquired from Region 3 was observed in Cluster 4, where no other Abuab were found. Given that Abuab is typically found in the Bicol Region (Department of Agriculture, 2018), Abuab_a from Region 3 is probably a misidentified abaca accession. In addition, a large portion of the percent shared alleles (-95%) shown in Figure 5 of Abuab_a came only from cluster 4, suggesting it is a different accession from the rest of the Abuab cultivar.

**Fig. 4:**
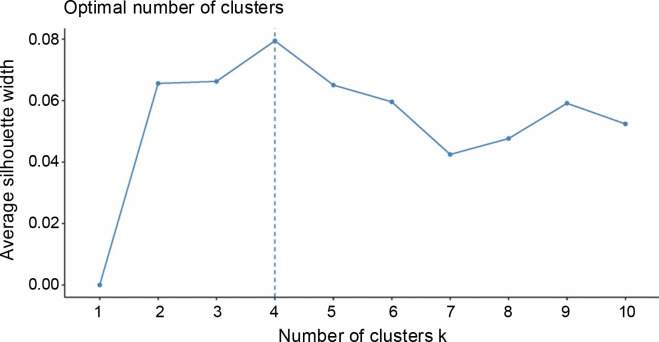
Number of ideal Kmean clustering of 93 abaca (*Musa textilis* Nee) collections according to the NbClust package of R studio

A total of 20 samples were grouped in cluster 3. Around 45% of this population originated from Region 9, while 30% came from Region 3, and 10% were from Region 8. This is the only cluster that deviated from the rest of the grouping since its members largely came from a region in Mindanao, indicating their genetic dissimilarity with the abaca accessions found in other regions. Three Lunhan and two Tangongon accessions are found here. The accessions with Jugbagon names are also placed in this cluster. This suggests that the cultivars found in this cluster are associated with one another and originated in region 9. Cluster 4 was composed of 23 abaca samples. The majority of accessions here are from Region 5 (39%), while Regions 6 and 3 shared 17% of the total number of abaca members in this group. Three Negro accessions, Abuab_a, and Inosa_b are the most notable members of this cluster, for they were separated from other abaca that share the same names. Clusters 1, 2, and 4 were dominated by abaca accessions from the Bicol region, and only cluster 3 was composed mostly of accessions from region 9 (-45%). Many of the accessions that have large similarities in their alleles were grouped in the same clusters, except for Kutay-Kutay, Laylay, and Puti which have a representative for every cluster. This cluster analysis is supported by the results of the NbClust package (Fig. 4), which suggests that the optimal *k* was four clusters. A 90% cophenetic correlation also proved the outcome of this study. It indicates a high goodness of fit of the generated unweighted neighbor-joining tree. The regions where the accessions were collected are important in this study due to abaca cultivars’ physical similarity with one another and their mode of distribution (using suckers), which can lead to misidentification or mislabeling of the cultivars.

### Bayesian model-based population structure

**Fig. 5:**
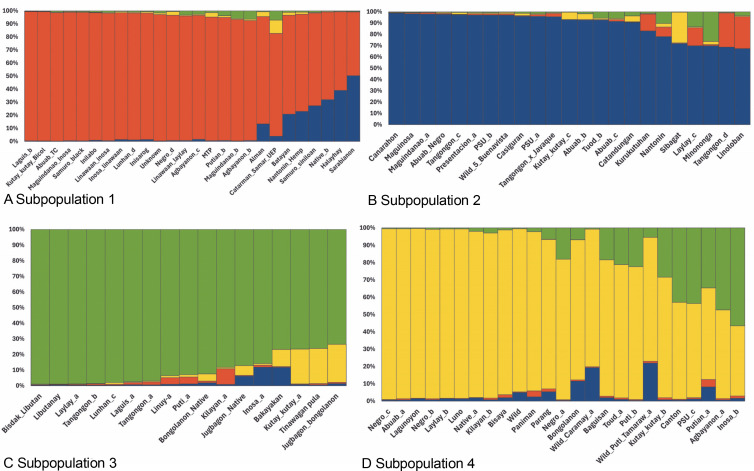
Four subpopulations generated by the STRUCTURE software v 2.3.4 for abaca (*Musa textilis* Nee) are based on the results of the 28 polymorphic genomic SSR markers; the red group (A – subpopulation 1), blue group (B – subpopulation 2), and yellow group (D – subpopulation 4) were composed mostly of abaca accessions from Region 5 while the majority of the green group © – subpopulation 3) were from Region 9

**Fig. 6:**
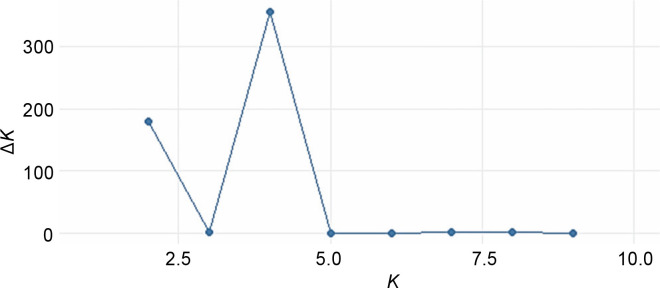
Best k clustering was generated by the Pophelper R package for population structure analysis of abaca (*Musa textilis* Nee)

The STRUCTURE software v.2.3.4 was used to further define the observed clustering of the 93 abaca accessions and confirm the results of the neighborjoining tree. The percentage of alleles shared between clusters is shown in Figure 5. The population structure corroborated with the cluster analysis generated by the DARwin software but with a few exceptions: Inosa_b, Lunhan_a, Putian_a, and Sarabianon regrouped into the adjacent subpopulation in this analysis. Subpopulation 1 (Fig. 5A) includes 25 abaca samples, where the inferred shared alleles ranged from 59.9 (Halayhay) to 99.1% (Kutay_kutay_Bicol). The large portion of the allelic distribution of Kutay_kutay_Bicol in subpopulation 1 indicates its genetic difference and uniqueness from other Kutay_kutay cultivars placed in other groups. Lina-waan_inosa and Inosa_Linawaan only differ by 0.4% of shared alleles, suggesting that they are the same accessions bearing interchanged names. In addition, these samples were also collected in the same region (Region 8). This also confirms the cultivars’ close association with the neighbor-joining tree (Fig. 3). Samuro_black and Samuro_siniloan share 98.6 and 70.8% of alleles from subpopulation 1, respectively, compared to other clusters, indicating they are related but not of the same accession. The samples Agbayanon_b and Agbayanon_c are both members of subpopulation 1 based on the -90% shared alleles but have varying percent alleles coming from other clusters, suggesting they are highly associated but not the same cultivars. Subpopulation 2 (Fig. 5B) is composed of 25 individuals. Sarabianon, which was placed in cluster 1 by the UNJ tree, was moved to subpopulation 2. This is because the alleles it shares with subpopulation 1 are almost at a 50/50 ratio: 48.6 (subpopulation 1) and 50.7% (subpopulation 2). Darwin may have placed it in cluster 1, ignoring the 2.1% difference in shared alleles. Using different diversity analysis programs is important to generate robust results in SSR markers. Both Presentation, a and b, are found in this subpopulation but bear different inferred ancestry or shared alleles (97.6 and 43.9%), indicating an association and molecular difference of the cultivars. All Abuab and PSU accessions here also shared more than 90% of subpopulation 2 but were a mix of different shared alleles; hence, they are highly associated but not the same accessions. Tangongon_c and the hybrid Tangongon x Javaque were more similar in shared alleles (-90%) than Tangongon_d (71%), indicating that the hybrid is more genetically similar to Tangongon_c than the Tangongon_d cultivar. Subpopulation 3 (Fig. 5C) is composed of 18 abaca accessions. Inosa_a and Inosa_b were collected from regions 13 and 8 and have different shared percent allele distribution in the subpopulation (85.9 and 58.5%, respectively), indicating low genetic similarity. In addition, Inosa_b, like Sarabianon, was also placed in the adjacent group (cluster 4) in the NJ tree. The Jugbagon_native (93.8%) and the hybrid Jugbagon_bongolanon (78%) are related according to their percent shared in subpopulation 3 but demonstrate genetic differences, the alleles shared by the Bongolanon parent with the hybrid is considered a factor for the allelic differences. Only Lunhan_a, which was previously grouped in a cluster of the NJ tree, got separated from Lunhan b and c cultivars and was placed in the adjacent subpopulation 4. In subpopulation 4 (Fig. 5D), the shared allelic percentage ranged from 47.3 (Putian_a) to 99% (Negro_c). Similar to the UNJ tree generated by DARwin, the three Negro accessions were grouped in this subpopulation. They possess varying percent alleles: Negro_a (80.2%), Negro_b (98%), and Negro_c (99%). This suggests that Negro_d, placed in subpopulation 1, possesses a different allelic distribution than the other Negro accessions. The measure of subpopulation differentiation obtained for the structure analysis was α = 0.0735 and average *F*_stp_ = 0.0815, both suggesting moderate genetic diversity. According to the Evanno method implemented in the Pophelper package, the optimum *K* clustering in this collection was four (Fig. 6), similar to the result obtained from the NbClust package (Fig. 4) generated for the UNJ tree.

### AMOVA and PCOA of the four abaca populations

**Fig. 7:**
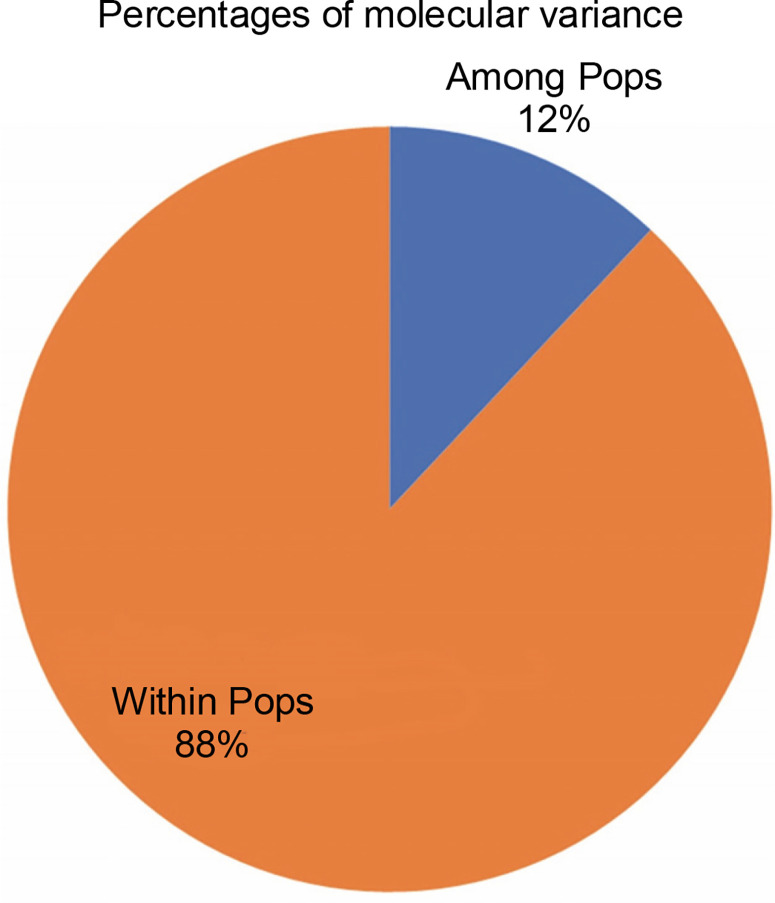
Percent allelic differentiation among and within the four subpopulations of abaca (*Musa textilis* Nee) based on the results of population structure analysis

**Fig. 8:**
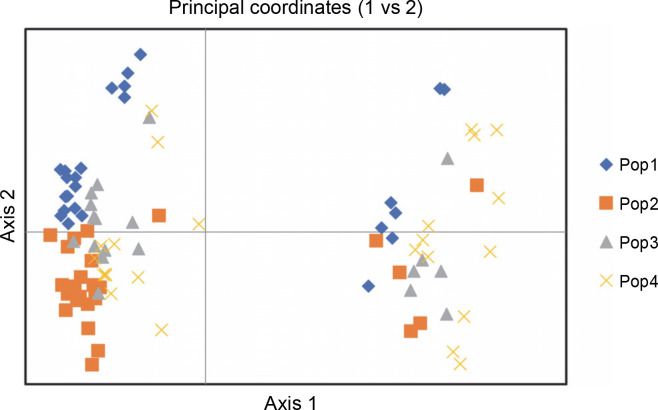
Principal coordinate analysis (PCoA) shows the relationships among the four abaca (*Musa textilis* Nee) subpopulations

Figure 7 exhibits the analysis of molecular variance (AMOVA). The variation among the four abaca subpopulations was 12%, while 88% allelic differentiation was observed within the population (Table 3). This result was different from Yllano et al. (2020) AMOVA results; nonetheless, both studies recorded low among-population molecular variation and high allelic distribution within populations. This shows that the individuals within the population have higher allelic variability than among populations. The high within-population differentiation may indicate a recent divergence of the abaca accession from their common ancestor. The three-axis generated by PCoA explains 24.63% (Table 4) of the cumulative variation in the abaca germplasm collection. The subpopulations were labeled with different shapes and colors to distinguish them from one another. Members of subpopulations 1–3, except for some data points, were together with their respective populations, while the accessions in subpopulation 4 were scattered all over the PCoA map (Fig. 8).

**Table 3:** The analysis of variance among and within subpopulations of abaca (*Musa textilis* Nee)

Source	df	SS	MS	Est. Var.	%
Among Pops	3	712.835	237.612	7.788	12
Within Pops	89	5094.401	57.240	57.240	88
Total	92	5807.237		65.028	100

**Table 4:** The principal coordinate analysis of abaca accessions (*Musa textilis* Nee)

Axis	1	2	3
%	12.09	7.24	5.30
Cum %	12.09	19.33	24.63

### Colinearity of the UNJ tree and model-based population structure

**Fig. 9:**
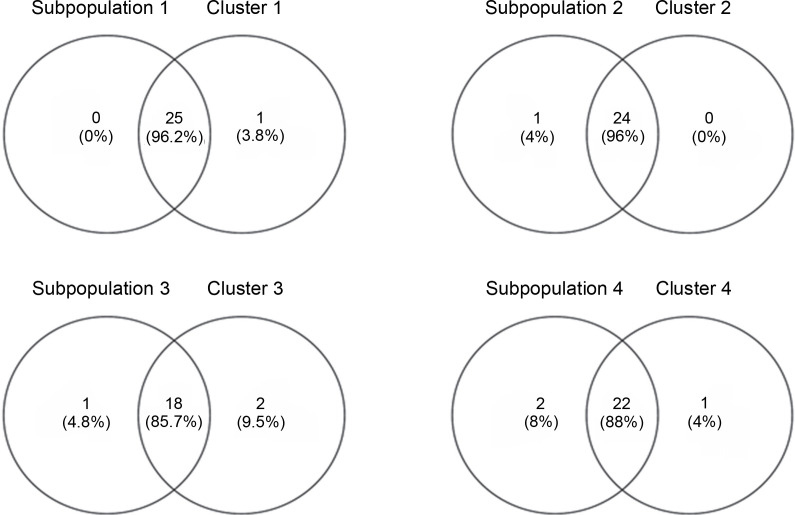
Venn diagram analysis of the inferred clusters of the STRUCTURE software and unweighted neighbour-joining tree of the DARwin program generated for abaca (*Musa textilis* Nee) shows 91% collinearity

Venny 2.1.0 was used to determine the similarity between the neighbor-joining tree and the subpopulations generated by DARwin and Structure software, respectively (Fig. 9). The percent similarity of each subpopulation with their respective clusters ranged from 85.7 to 96.2%, averaging 91% accessions shared between the populations built by the two software. This shows high corroboration among the generated groupings. Sarabianon was found exclusive to cluster 1 of the UNJ tree but grouped to subpopulation 2 by the Structure software. Populations 3 and 4 and clusters 3 and 4 differed by three accessions: namely, Inosa_b, Lunhan_a, and Putian_a. The high percentage of similarity between the clustering systems exhibits a high accuracy of the developed polymorphic markers.

### Cross-transferability to other Musa species

**Fig. 10:**
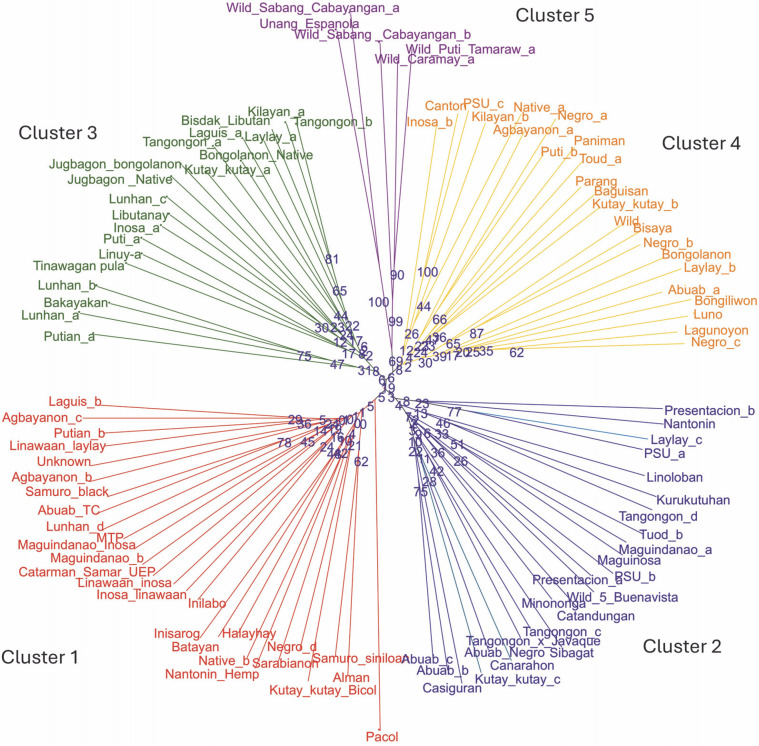
Unweighted neighbor-joining tree was generated for the 99 Musa collection, to test the cross-transferability of the newly developed molecular markers with other Musa

**Fig. 11:**
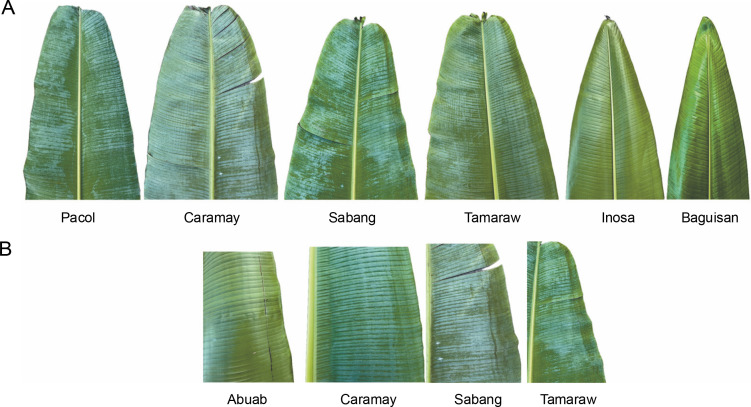
Member of the fifth cluster from the UNJ tree; (A) powdery leaf blade underside of the members of the fifth cluster (Caramay, Sabang, Tamaraw) is similar to Pacol compared with the glossy underside of the Inosa and Baguisan leaf blades, (B) red or green line found on the right side of the abaca leaf blade was a shared character by the fifth cluster with the abuab

The 28 newly developed genomic SSR primers were also tested for cross-transferability to other *Musa* species (Fig. 10). The groupings generated from this genetic analysis agree with the previous UNJ tree, except that an additional cluster was formed. The members of the fifth cluster exhibit some morphological characteristics that were a mixture of the botanical descriptions for abaca and banana. In Figure 11, the underside of the leaf blade of the fifth cluster members was powdery and their leaf shape was similar to Pacol (*M. balbisiana* Colla), however, the distinct red or green line was only found on the underside of the abaca leaf blade was also observed in the fifth cluster’s accessions. Pacol is a well-known wild *M. balbisiana* Colla accession that showed resistance to the bunchy top virus, it exhibits a wider leaf blade with a powdery underside and overlapping fruit bracts. The markers designed in this study align with all 11 chromosomes of *M. balbisiana* Colla which is the reason for Pacol grouping with cluster 1. However, there is an obvious difference in the branching and edge length of Pacol to the rest of the accessions in the said cluster indicating genetic differences with the abaca accessions. The result of this transferability shows the specificity of the 28 polymorphic markers to the abaca genome and their capacity to delineate other *Musa* species. The construction of this tree is supported by the fit criterion of the cophenetic correlation of 91%.

## Discussion

Our study is the first report on the efficiency of genome-wide molecular markers based on the whole genome assembly of Abaca var. Abuab. The goal of this study is to develop accurate, inexpensive, and robust primers that can be used for diversity analysis, variety verification, candidate gene association mapping, and hybrid. Our results add to the repository of comprehensive reports on the genetic variability of the abaca collection using SSR markers. The discriminating power of genomic SSRs has been shown in complex genomes like bread wheat (Ahmed et al., 2020) and banana (Biswas et al., 2020), as well as gene mapping for diseases such as stripe rust in wheat (Zhou et al., 2024), indicating the robustness of these markers for assessing genetic variation. This molecular marker system is not only used in the determination and conservation of true genetic resources but also plant breeding. The marker RM16089 was linked to the late-maturing gene *Hd16* in rice (Tomita et al., 2022). The BNL252, NAU3424, NAU3324, and CGR5202 SSR markers were highly associated with fiber quality traits of the cotton variety *G. barbadense* (Ma et al., 2017). Twenty-one SSR markers were found associated with different agronomic traits of maize, such as kernel weight, plant architecture, ear length, and thickness. These studies prove that SSR markers are useful in crop improvement activities in maize (Park et al., 2015).

The identified 50 genomic molecular markers represent scaffolds that aligned to nine of the eleven chromosomes of *M. balbisiana* Colla indicating their genome-wide distribution in the abaca genome. The polymorphism information content (PIC) value of 0 indicates band monomorphism, while values closer to 1 mean high polymorphism (Hildebrand, 1992). The PIC values of markers are further categorized as highly informative (≥ 0.5), moderately informative (0.50–0.25), or least informative (≤ 0.25) (Botstein et al., 1980). A total of 28 markers out of the newly developed 50 primers showed high polymorphism, ranging from 0.49 (> MK42523) to 0.91 (> MK2915). The polymorphic information content in this study averages 0.78 (> 0.5), showing high allelic diversity among the individual abaca accessions. This average value was also higher than the 0.61 and 0.56 PIC scores obtained from the studies of Boguero et al. (2016) and Yllano et al. (2020), respectively. They both worked on the diversity of abaca however, they used markers based on banana and other genera that have low specificity for abaca resulting in the generation of many clusters. The differences in the genetic composition and number of samples used affect the computed PIC values (Biswas et al., 2020). The 28 highly polymorphic abacabased markers in this study are additional molecular markers to the six banana-based primers previously used on abaca. A total of 14 SSR markers could differentiate and group Ethiopian bananas into three clusters. The markers used show a high average PIC value of 0.82 (Workneh et al., 2022). Similarly, 14 polymorphic primers were able to genotype *Musa balbisiana* Colla cultivars generating three clusters (Doloiras et al., 2018). The 12 polymorphic SSR primers were also used to determine the clone identity of the banana. Some of these molecular markers generated unique fingerprints for Ardhapuri banana cultivars (Hinge et al., 2022). This showed that even at low numbers, our abaca genome-specific SSR markers are capable of distinguishing individuals and closely related species. Prioritizing only the unique accessions in the germplasm can help in the conservation and proper utilization of true abaca-registered and traditional varieties, especially in germplasm preservation and abaca breeding.

The average genetic diversity computed was 0.428, lower than the observed heterozygosity obtained by Yllano et al. (2020) and Boguero et al. (2016) at 0.52 and 0.92, respectively. This indicates that our collection has moderate heterogeneity. Our results are supported by the fact that the asexual propagation of abaca resulted in its narrow genetic base (Purwati et al., 2018). The mean *F*_stp_ value of 0.0815 computed by the Hierfstat package in R indicates moderate diversity between subpopulations. The *F*_st_ value shows the allele frequency distribution among populations. The *F*_st_ value > 0.25 indicates high genetic differentiation suggesting the accessions came from different species while closer to 0 indicates little genetic differentiation. The intermediate or moderate differentiation lies between 0.05 and 0.15 *F*_st_ values (Li et al., 2014; Luo et al., 2019). The mean value of alpha obtained from population analysis was α = 0.0735. The relatively small alpha values mean that a few members of the population were admixed, and the accessions originated from one ancestor. The alpha value approaching zero indicates that the accessions are from separate populations, while if it is greater than 1, it means that the members are highly admixed and originated from multiple sources. This agrees with the computed population heterozygosity of 0.428 and the AMOVA results, where greater within population variation (88%) was found.

Boguero et al. (2016) looked at the association of six SSR primers with bunchy-top disease and generated 10 groups using cluster analysis, while Yllano et al. (2020) used banana-based microsatellite markers that produced 9 clusters in the neighbor-joining tree. The high allelic distribution of SSR markers tends to produce increased genetic variation even among closely related species (Aiello et al., 2020) resulting in more clusters. The nature of populations and molecular markers also cause varying population structures (Zhu et al., 2016). The specificity of the 28 genomic markers used for abaca accessions in this study resulted in only four inferred populations. Two different software programs (DARwin and STRUCTURE v. 2.3.4) generated clusters and populations that were 91% similar according to the Venn diagram analysis. This percent collinearity is considered high because a percent similarity of 55% between the clusters and population exhibited high support in a genetic study of rice germplasm (Singh et al., 2016). The four subpopulations identified by DARwin showed that some samples bearing similar names grouped with varying clusters. The population structure analysis, on the other hand, revealed the allelic distribution per accession and revealed that the accession that clustered differently by DARwin possessed allele distribution bordering two subpopulations. The inferred clusters also show that the accessions with inverted names are identical varieties. This shows that the newly developed genome-wide SSR markers specificity of markers dedicated to genetic diversity analysis, variety identification, and association mapping for abaca. These newly developed polymorphic markers will assist the breeders in planning hybridization activities and selecting the true abaca varieties that can be used in crop improvement given that the abaca population has moderate genetic diversity.

The cross-transferability of markers based on the genomes of other species has been done in abaca (Yllano et al., 2020), asparagus (Geetha and Siril, 2022), and fennel (Aiello et al., 2020). For the fennel, only 23% of carrot SSR markers were transferable; therefore, they concluded that the transferability of primers across genera is challenging (Aiello et al., 2020). The 28 markers used in this study were able to identify other *Musa* species that share physical similarities with abaca and place them in an additional cluster in the UNJ tree. In addition, the association of Pacol with Cluster 1 shows that abaca and *M. balbisiana* Colla share genetic similarities but are not the same, for they differ in branch length and placement in Cluster 1. This shows that the markers can delineate abaca from their close relative *Musa* species. Oftentimes, farmers label the same variety with different names (Halos, 2008). This is perhaps the case with some of the abaca cultivars in this study. The accessions were given different names by farmers but are the same, and vice versa. This is due to the confusing morphology that the abaca shares with one another and other *Musa* species. The 28 genomic markers were able to fingerprint the members of the abaca germplasm collection.

The percent of molecular variance found in the collection was 12% among populations and 88% within populations. These results corroborate with Yllano et al. (2020), who generated 5% among population variance and 95% within populations. The low percent variation among the population was also observed in rice (Singh et al., 2016) and grain amaranth (Vats, 2023). Some of the grouping established in this study corroborates with the clusters generated by SNP data of Barbosa et al. (2023) where Tangongon, Puti, and Kutay_kutay_a are grouped in the same cluster (cluster 3). Abuab_a, Luno, and Inosa_b are members of cluster 4. This proves that developed molecular markers can fully characterize the abaca populations. Our primers are hypervariable, widely distributed, and informative tools; hence it became our go-to marker of choice.

## Conclusion

The improvement in genotyping and variety identification of abaca eases conventional breeding techniques and maximizes resources for germplasm conservation. The intense selection bias towards elite individuals results in the neglect of the diverse genetic sources available in the field. This study increased specificity in genotyping physically similar *Musa* collections through the use of polymorphic SSR loci (-78% PIC). Accessions that shared the same names were grouped in clusters using two different diversity analysis tools (DARwin and Structure v2.3.4). Our study is a thorough examination of the extent of the relatedness of abaca and DNA fingerprints using genome-wide molecular markers. The abaca germplasm collection used in this study was collected from ten regions in the Philippines, and many of them shared similar or same names. Using molecular markers, especially these genome-specific markers, saves time in fingerprinting, variety verification, and candidate gene linkage mapping of economically important traits. This study is an in-depth look at the genetic variability among the members of the abaca gene bank.

## Supplementary Material

Newly developed genomic SSR markers revealed the population structure and genetic characteristics of abaca (*Musa textilis* Nee)

## References

[cit0001] Ahmed H.G.M.D., Kashif M., Rashid M.A.R., Sajjad M., Zeng Y. (2020) Genome-wide diversity in bread wheat evaluated by SSR markers. Intl. J. Agric. Biol. 24: 263–272. 10.17957/IJAB/15.143

[cit0002] Aiello D., Ferradini N., Torelli L., Volpi C., Lambalk J., Russi L., Albertini E. (2020) Evaluation of cross-species transferability of SSR markers in Foeniculum vulgare. Plants (Basel) 9(2): 175. 10.3390/plants9020175 32024130 PMC7076658

[cit0003] Barbosa C.F.C., Asunto J.C., Koh R.B.L., Santos D.M.C., Zhang D., Cao E.P., Galvez L.C. (2023) Genome-wide SNP and indel discovery in Abaca (Musa textilis Née) and among other Musa spp. for abaca genetic resources management. Curr. Issues Mol. Biol. 45(7): 5776–5797. 10.3390/cimb45070365 37504281 PMC10377871

[cit0004] Bhattarai G., Shi A., Kandel D.R., Solis-Gracia N., da Silva J.A., Avila C.A. (2021) Genome-wide simple sequence repeats (SSR) markers discovered from whole-genome sequence comparisons of multiple spinach accessions. Sci. Rep. 11: 9999. 10.1038/s41598-021-89473-0 33976335 PMC8113571

[cit0005] Biswas M.K., Bagchi M., Biswas D., Harikrishna J.A., Liu Y., Li C., Sheng O., Mayer C., Yi G., Deng G. (2020) Genome-wide novel genic microsatellite marker resource development and validation for genetic diversity and population structure analysis of banana. Genes (Basel) 11(12): 1479. 10.3390/genes11121479 33317074 PMC7763637

[cit0006] Boguero A.P., Parducho M.A., Mendoza M.R.D.R., Abustan M.A.M., Lalusin A.G. (2016) Molecular screening of abaca (M. textilis Nee) accessions using microsatellite markers associated with resistance to bunchy top disease. Philippine J. Crop Sci. 41(2): 13–19.

[cit0007] Botstein D., White R.L., Skolnick M., Davis R.W. (1980) Construction of a genetic linkage map in man using restriction fragment length polymorphisms. Am. J. Hum. Genet. 32: 314–331. 6247908 PMC1686077

[cit0008] Choudhury D.R., Kumar R., Maurya A., Semwal D.P., Rathi R.S., Gautam R.K., Trivedi A.K., Bishnoi S.K., Ahlawat S.P., Singh K., Singh N., Singh R. (2023) SSR and SNP marker-based investigation of Indian rice landraces in relation to their genetic diversity, population structure, and geographical isolation. Agriculture 13: 823. 10.3390/agriculture13040823

[cit0009] Damo J.L.C. (2012) *Molecular characterization of selected BC2F1 individuals of abaca (Musa textilis Nee) for tensile strength using microsatellite markers*. Thesis manuscript. University of the Philippines Los Baños, Laguna.

[cit0010] Doloiras-Larano A.R., Garcia R.N., Sandoval C.M., Lalusin A.G., Gueco L.S., Huelgas V.C., Tecson-Mendoza E.M. (2018) DNA fingerprinting and genetic diversity analysis of Philippine Saba and other cultivars of Musa balbisiana Colla using simple sequence repeat markers. Philippine J. Crop Sci. 41(2): 13–19. https://www.cabi.org/gara/FullTextPDF/2018/20183300820.pdf

[cit0011] Ebert A.W., Engels J.M.M., Schafleitner R., Hintum Tv., Mwila G. (2023) Critical review of the increasing complexity of access and benefit-sharing policies of genetic resources for genebank curators and plant breeders – a public and private sector perspective. Plants 12: 2992. 10.3390/plants12162992 37631201 PMC10459714

[cit0012] Fandade V., Singh P., Singh D., Sharma H., Thakur G., Saini S., et al. (2023) Genome-wide identification of microsatellites for mapping, genetic diversity and cross-transferability in wheat (Triticum spp.). Gene 896: 148039. 10.1016/j.gene.2023.148039 38036075

[cit0013] Francis R.M. (2017) Pophelper: an R package and web app to analyze and visualize population structure. Mol. Ecol. Resour. 17: 27–32. 10.1111/1755-0998.12509 26850166

[cit0014] Galvez L.C., Koh R.B.L., Barbosa C.F.C., Asunto J.C., Catalla J.L., Atienza R.G., et al. (2021) Sequencing and de novo assembly of abaca (Musa textilis Née) var. Abuab genome. Genes (Basel) 12(8): 1202. 10.3390/genes12081202 34440376 PMC8392402

[cit0015] García C., Guichoux E., Hampe, A. (2018) A comparative analysis between SNPs and SSRs to investigate genetic variation in a juniper species (Juniperus phoenicea ssp. turbinata). Tree Genet. Genomes 14: 87. 10.1007/s11295-018-1301-x

[cit0016] Geetha C.M., Siril E.A. (2022) Cross-species transferability of genomic SSR markers and genetic diversity among Asparagus racemosus. Plant Gene. 31: 100361. 10.1016/j.plgene.2022.100361

[cit0017] Halos S.C. (2008) The Abaca. Department of Agriculture-Biotechnology Program Office. ISBN 978-971-94191-0-5

[cit0018] Hildebrand C.E., Torney D.C., Wagner R.P. (1992) *Informativeness of polymorphic DNA markers*. https://sgp.fas.org/othergov/doe/lanl/pubs/00326695.pdf

[cit0019] Hinge V.R., Shaikh I.M., Chavhan R.L. Deshmukh A.S., Shelake R.M., Ghuge S.A., Dethe A.M., Suprasanna P., Kadam U.S. (2022) Assessment of genetic diversity and volatile content of commercially grown banana (Musa spp.) cultivars. Sci. Rep. 12: 7979. 10.1038/s41598-022-11992-1 35562398 PMC9106755

[cit0020] Itoo H., Shah R.A., Qurat S., Jeelani A., Khursheed S., Bhat Z.A., Mir M.A., Rather G.H., Zagar S.M., Shah M.D., Padder B.A. (2023) Genome-wide characterization and development of SSR markers for genetic diversity analysis in northwestern Himalayas Walnut (Juglans regia L.). Biotech 13: 136. 10.1007/s13205-023-03563-6 PMC10130282 37124992

[cit0021] Kaldate R., Rana M., Sharma V., Hirakawa H., Kumar R., Singh G., et al. (2017) Development of genome-wide SSR markers in horsegram and their use for genetic diversity and cross transferability analysis. Mol. Breed. 37: 103. 10.1007/s11032-017-0701-1

[cit0022] Lalusin A. (2010) *Abaca breeding for a more reliable Philippine Abaca Industry*. Annual BSP UP Professorial Chair Lectures. BangkoSentralnf Pilipinas. Malate, Manila.

[cit0023] Lazar I., Lazar Jr I., (2023) *GelAnalyzer 23.1.1* (available at www.gelanalyzer.com)

[cit0024] Li F.P., Lee Y.S., Kwon S.W., Li G., Park Y.J. (2014) Analysis of genetic diversity trait correlations among Korean landrace rice (Oryza sativa L). Genet. Mol. Res. 13(3): 6316–6331. http://www.funpecrp.com.br/gmr/year2014/vol13-3/pdf/gmr3271.pdf 24782213 10.4238/2014.April.14.12

[cit0025] Liu J., Magige E.A., Fan P.Z. Wambulwa M.C., Luo Y.H., Qi H.L., Gao L.M., Milne R. (2023) Genetic imprints of grafting in wild iron walnut populations in southwestern China. BMC Plant Biol. 23: 423. 10.1186/s12870-023-04428-z 37700228 PMC10498525

[cit0026] Liu F., Movahedi A., Yang W., Xu D., Jiang C., Xie J., Zhang Y. (2021) The complete chloroplast genome and characteristics analysis of Musa basjoo Siebold. Mol. Biol. Rep. 48(11): 7113–7125. 34541615 10.1007/s11033-021-06702-5

[cit0027] Liu S., Feuerstein U., Luesink W., Schulze S., Asp T., Studer B., Becker H.C., Dehmer K.J. (2018) DArT, SNP, and SSR analyses of genetic diversity in Lolium perenne L. using bulk sampling. BMC Genet. 19: 10. 10.1186/s12863-017-0589-0 29357832 PMC5778656

[cit0028] Luo Z., Brock J., Dyer J.M., Kutchan T., Schachtman D., Augustin M., Ge Y., Fahlgren N., Abdel-Haleem H. (2019) Genetic diversity and population structure of a Camelina sativa Spring Panel. Front. Plant Sci. 10: 184. 10.3389/fpls.2019.00184 30842785 PMC6391347

[cit0029] Martin G., Baurens F.C., Droc G., Rouard M., Cenci A., Kilian A., Hastie A., Doležel J., Aury J.M., Alberti A., Carreel F., D’hont A. (2016) Improvement of the banana “Musa acuminata” reference sequence using NGS data and semi-automated bioinformatics methods. BMC Genom. 17: 243. 10.1186/s12864-016-2579-4 PMC4793746 26984673

[cit0030] Ma Q., Zhao J., Lin H., Ning X., Liu P., Deng F., Si A., Lian, J. (2017) Association between SSR markers and fibre traits in sea island cotton (Gossypium barbadense) germplasm resources. J. Genet. 96: e55–e63. http://www.ias.ac.in/jgenet/OnlineResources/96/e55.pdf 29321342 10.1007/s12041-017-0849-9

[cit0031] Muncada G.P. (2018) *Next generation sequencing transcriptome analysis of abaca bunchy top virus resistance and differential expression of EIF4G gene in abuab, pacol, and BC2 Hybrid*. PhD Manuscript.

[cit0032] Niu Y.F., Gao C.W., Liu J. (2018) The complete chloroplast genome sequence of wild banana, Musa balbisiana variety ’Pisang Klutuk Wulung’ (Musaceae). Mitochondrial DNA Part B 3(1): 460–461. 10.1080/23802359.2018.1462123 33490514 PMC7800968

[cit0033] Oliveros J.C. (2007) *Venny. An interactive tool for comparing lists with Venn’s diagrams*. https://bioinfogp.cnb.csic.es/tools/venny/index.html

[cit0034] Pabro L.J. (2012) *Morphological characterization and DNA fingerprinting of abaca (M. textilis Nee) for bunchy top virus resistance using molecular markers*. MSc manuscript. University of the Philippines Los Baños, Laguna.

[cit0035] Palao C.D.O. (2019) *Characterization and development of microsatellite markers from RNA-seq data of abaca (Musa textilis Nee)*. PhD dissertation. University of the Philippines Los Baños, Laguna.

[cit0036] Pan G., Chen A., Li J. et al. (2020) Genome-wide development of simple sequence repeats database for flax (Linum usitatissimum L.) and its use for genetic diversity assessment. Genet. Resour. Crop Evol. 67: 865–874. 10.1007/s10722-020-00882-y

[cit0037] Park J.Y., Ramekar R.V., Sa K.J., Lee, J.K. (2015) Genetic diversity, population structure, and association mapping of biomass traits in maize with simple sequence repeat markers. Genes & Genomics 37(8): 725–735.

[cit0038] Perrier X., Flori A., Bonnot, F. (2003) Data analysis methods. [in:] *Genetic diversity of cultivated tropical plants*. Ed. Hamon P., Seguin M., Perrier X., Glaszmann J.C. Ed., Enfield, Science Publishers. Montpellier: 43–76.

[cit0039] Perrier X., Jacquemoud-Collet J.P. (2006) *DARwin software*. https://darwin.cirad.fr/

[cit0040] Peakall R., Smouse P.E. (2012) GenAlEx 6.5: genetic analysis in Excel. Population genetic software for teaching and research-an update. Bioinformatics 28: 2537–2539. 22820204 10.1093/bioinformatics/bts460 PMC3463245

[cit0041] Pritchard J.K., Stephens M., Rosenberg N.A., Donnelly P. (2000) Association mapping in structured populations. Am. J. Hum. Genet. 67: 170–181. 10.1086/302959 10827107 PMC1287075

[cit0042] Puwarti R.D., Parnidi, Setyu-Budi U., Herwati A. (2018) Identification of high yield clones of abaca (Musa textilis Nee) mutants based on morphological characters. iVolga Press. Biotika 23(4): 3–8.

[cit0043] Reamillo M.C.S. (2018) *Genetic characterization of backcross progenies BC1, BC2 from Musa textilis Nee (Abuab) x Musa balbisiana colla (Pacol) through transcriptomic analysis*. PhD manuscript. University of the Philippine Los Baños Laguna.

[cit0044] Salgotra R.K., Chauhan B.S. (2023) Genetic diversity, conservation, and utilization of plant genetic resources. Genes 14: 174. 10.3390/genes14010174. 36672915 PMC9859222

[cit0045] Sandoval C.M.C. (2011) Molecular and biochemical studies of cassava (Manihot esculenta Crantz): seed coat phenolics, seed storage proteins and DNA fingerprinting for storage root color, hydrocyanic acid (HCN) content and bacterial blight resistance. Graduate Student's Output. 345. https://www.ukdr.uplb.edu.ph/etd-grad/345

[cit0046] Singh J., Sharma A., Sharma V., Gaikwad P.N., Sidhu G.S., Kaur G., Kaur N., Jindal T., Chhuneja P., Rattanpal H.S. (2023) Comprehensive genome-wide identification and transferability of chromosome-specific highly variable microsatellite markers from citrus species. Sci. Rep. 13(1): 10919. 10.1038/s41598-023-37024-0 37407627 PMC10322976

[cit0047] Singh N., Choudhury D.R., Singh A.K., Kumar S., Srinivasan K., Tyagi R.K., Singh N.K., Singh R. (2013) Comparison of SSR and SNP markers in estimation of genetic diversity and population structure of Indian rice varieties. PLoS One 8(12): e84136. 10.1371/journal.pone.0084136 24367635 PMC3868579

[cit0048] Tamazian G., Dobrynin P., Krasheninnikova K., Komissarov A., Koepfli K.P., O’Brien S.J. (2016) Chromosomer: a reference-based genome arrangement tool for producing draft chromosome sequences. Gigascience 5(1): 38. 10.1186/s13742-016-0141-6 27549770 PMC4994284

[cit0049] Tomita M., Tokuyama R., Matsumoto S., Ishii K. (2022) Whole-genome sequencing revealed a late-maturing isogenic rice koshihikari integrated with Hd16 gene derived from an ise shrine mutant. Int. J. Genomics 6: 4565977. 10.1155/2022/4565977 PMC8758330 35036423

[cit0050] Vats G., Das D., Gupta R., Singh A., Maurya A., Rajkumar S., et al. (2023) Validation of genome-wide SSR markers developed for genetic diversity and population structure study in grain amaranth (Amaranthus hypochondriacus). Agriculture 13: 431. 10.3390/agriculture13020431

[cit0051] Vilela J., Diaz M.G., Ocampo E.T., Lalusin A.G., Laurena A.C. (2015) Molecular analyses of the abaca (Musa textilis nee cv. Abuab) filtered-genome using next generation sequencing and sanger-based sequencing. Trans. Natl Acad. Sci. Technol. 37(1): 33–35.

[cit0052] Wang Z., Miao H., Liu J., Xu B., Yao X., Xu C., et al. (2019) Musa balbisiana genome reveals subgenome evolution and functional divergence. Nat Plants. 5(8): 810–821. 10.1038/s41477-019-0452-6 31308504 PMC6784884

[cit0053] Wang X., Wang L. (2016) GMATA: An integrated software package for genome-scale SSR mining, marker development and viewing. Sec. Plant Genet. Genom. 7. 10.3389/fpls.2016.01350 PMC5020087 27679641

[cit0054] Workneh S.T., Alemu S.K., Olani G., Debebe A., Berhanu B., Dagnew A., Assefa W. (2022) Molecular characterization of banana genotypes by SSR markers. AJPS 16(9): 258–269. 10.5897/AJPS2022.2268

[cit0055] Wu X., Hornyik C., Bayer M., Marshall D., Waugh R., Zhang R. (2014) In silico identification and characterization of conserved plant microRNAs in barley. Central Eur. J. Biol. 9(9): 841–852. 10.2478/s11535-014-0308-z

[cit0056] Wu W., Yang Y.L., He W.M., Rouard M., Li W.M., Xu M., Roux N., Ge X.J. (2016) Whole genome sequencing of a banana wild relative Musa itinerans provides insights into lineagespecific diversification of the Musa genus. Sci. Rep. 6: 31586. 10.1038/srep31586 27531320 PMC4987669

[cit0057] Yllano O.B., Diaz M.G.Q., Lalusin A.G., Laurena A.C., Tecson-Mendoza E.M. (2020) Genetic analyses of abaca (Musa textilis Née) germplasm from its primary center of origin, the Philippines, using simple sequence repeat (SSR) markers. Philipp. Agric. Sci. 103: 311–321.

[cit0058] Zhang J., Yang J., Lv Y., Zhang X., Xia C., Zhao H., Wen C. (2023) Genetic diversity analysis and variety identification using SSR and SNP markers in melon. BMC Plant Biol. 23: 39. 10.1186/s12870-023-04056-7 36650465 PMC9847184

[cit0059] Zhou X., Wang Y., Luo Y., Shuai J., Jia G., Chen H., Li X., Huang K., Yang S., Wang M., Ren Y., Li G., Chen X. (2024) Genome-wide mapping of quantitative trait loci conferring resistance to stripe rust in spring wheat line PI 660072. Res. Square 10.21203/rs.3.rs-3752526/v1 39443304

[cit0060] Zhu H., Song P., Koo D.H., Guo L., Li Y., Sun S., Weng Y., Yang L. (2016) Genome wide characterization of simple sequence repeats in watermelon genome and their application in comparative mapping and genetic diversity analysis. BMC Genomics 17: 557. 10.1186/s12864-016-2870-4 27495254 PMC4974753

