# Supplementary material for: Newly developed genomic SSR markers revealed the population structure and genetic characteristics of abaca (Musa textilis Nee)
Source: BioTechnologia (Pozn). 2024 Dec 19;105(4):337–53. doi: 10.5114/bta.2024.145255 (PMC11748220; doi:10.5114/bta.2024.145255)
Supplement: Newly developed genomic SSR markers revealed the population structure and genetic characteristics of abaca (Musa textilis Nee) [file BTA-105-4-55194-s001.pdf]

BioTechnologia vol. 105 (4) • 2024
Journal of Biotechnology, Computational Biology and Bionanotechnology SUPPLEMENTARY MATERIALS
https://doi.org/10.5114/bta.2024.145255
CC BY 4.0

Newly developed genomic SSR markers revealed the population structure and genetic characteristics of abaca (*Musa textilis* Nee)

MARIECRIS RIZALYN R. MENDOZA [1]*, ANTONIO C. LAURENA [2], MARIA GENALEEN Q. DIAZ [3], EUREKA TERESA M. OCAMPO [2], TONETTE P. LAUDE [2], ANTONIO G. LALUSIN [2]

[1] Institute of Plant Breeding, College of Agriculture and Food Science, University of the Philippines Los Baños, Philippines
[2] Institute of Crop Science, College of Agriculture and Food Science, University of the Philippines Los Baños, Philippines
[3] Institute of Biological Sciences, College of Arts and Sciences, University of the Philippines Los Baños, Philippines

Received: 1 April 2024; revised: 10 September 2024; accepted: 24 October 2024

Abstract

Abaca (*Musa textilis* Nee) is the primary source of manila hemp fiber, a vital industrial product for the country. Previous studies have relied on molecular markers designed for other *Musa* species or distant genera like rice, limiting accurate genetic characterization and germplasm conservation. To address this, we developed 50 genome-specific molecular markers based on the recently released whole genome sequence assembly of Abaca var. *Abuab* by Galvez et al. (2021). Among these markers, 28 showed high polymorphism, with an average PIC value of 0.78. Population analysis revealed a heterozygosity of 0.428, indicating moderate genetic diversity, supported by an alpha value of 0.0735 and an F_{stp} value of 0.0815, which suggests moderate genetic differentiation among abaca accessions. Cluster analyses, generated by DARwin and STRUCTURE software with 91% similarity, identified four clusters. The new markers were also able to distinguish six *Musa* accessions exhibiting morphological traits of both abaca and banana. Discrepancies in sample identification due to identical or inverted names were resolved using population structure analysis. Molecular variance analysis showed a 12% variance among the four abaca subpopulations and 88% within populations, suggesting recent divergence. Our study highlights the diversity, identity, and genetic variation within the abaca collection using accurate, robust, cost-effective, and computationally simple genome-specific markers. These markers are pivotal for genetic studies of abaca, including trait-marker mapping and the differentiation of accessions even in the juvenile stage, when phenotypic differences may be subtle.

Key words: abaca, AMOVA, DARwin, genetic diversity, genomic SSR markers, population structure

Supplementary Table. The list of the *Musa* accessions analyzed in the study using newly developed molecular markers

Sample	Location	Sample	Location
Putian_*a*	Region IX	Kutay_kutay_*c*	Region IV-B
Lunhan_*a*	Region IX	Casiguran	Region III
Bakayakan	Region IX	Canarahon	Region V
Lunhan_*b*	Region IX	Catandungan	Region V
Lunhan_*c*	Region IX	Abuab_Negro	Region V
Jugbagon _Native	Region IX	Abuab_*b*	Region V
Jugbagon_bongolanon	Region IX	Abuab_*c*	Region V
Kutay_kutay_*a*	Region IX	Tuod_*b*	Region XII
Inosa_*a*	Region XIII	Tangongon_x_Javaque	Region XII

* Corresponding author: Institute of Plant Breeding, College of Agriculture and Food Science, University of the Philippines Los Baños, Philippines; e-mail: mdmendoza11@up.edu.ph

Table continued

Sample	Location	Sample	Location
Laylay_*a*	Region XIII	Maguindanao_*a*	Region XII
Laguis_*a*	Region XIII	Maguinosa	Region XII
Puti_*a*	Region VII	Tangongon_*c*	Region XII
Tangongon_*a*	Region IX	PSU_*b*	Region IV-B
Bisdak_Libutan	Region XIII	Minononga	Region V
Tangongon_*b*	Region XIII	PSU_*a*	Region IV-B
Kilayan_*a*	Region XIII	Presentacion_*a*	Region V
Linuy-a	Region VIII	Wild_5_Buenavista	Region IV-B
Bongolanon_Native	Region VII	Kurukutuhan	Region V
Libutanay	Region VIII	Linoloban	Region V
Inosa_b	Region VIII	Nantonin	CAR
Agbayanon_*a*	Region VI	Laylay_*c*	Region V
Tinawagan pula	Region V	Presentacion_*b*	Region IV-B
Paniman	Region V	Tangongon_*d*	Region XIII
Kutay_kutay_*b*	Region V	Sarabianon	Region XIII
Canton	Region V	Batayan	Region XIII
PSU_*c*	Region IV-B	Halayhay	Region XIII
Puti_*b*	Region V	Native_*b*	Region VI
Toud_*a*	Region V	Nantonin_Hemp	CAR
Baguisan	Region V	Linawaan_inosa	Region VIII
Negro_*a*	Region V	Inosa_linawaan	Region VIII
Parang	Region V	Laguis_*b*	Region XIII
Kilayan_*b*	Region VII	Inisarog	Region V
Native_*a*	Region VI	Unknown	Region V
Negro_*b*	Region VI	Negro_*d*	Region VI
Bisaya	Region VI	Catarman_Samar_UEP	Region VIII
Wild	Region III	Maguindanao_*b*	Region XII
Abuab_*a*	Region III	Agbayanon_b	Region VI
Lagunoyon	Region III	MTP	Region V
Luno	CAR	Agbayanon_c	Region VI
Negro_*c*	Region III	Linawaan_laylay	Region VIII
Laylay_*b*	CAR	Abuab_TC	Region V
Unang_Espanola	Region IV-B	Samuro_black	Region V
Wild_Caramay_*a*	Region IV-B	Lunhan_d	Region IX
Bongolanon	Region VII	Maguindanao_Inosa	Region XII
Wild_Puti_Tamaraw_*a*	Region IV-B	Putian_*b*	Socsargen
Bongiliwon	Region V	Kutay_kutay_Bicol	Region V
Wild_Sabang_Cabayangan_*a*	Region IV-B	Inilabo	Region V
Wild_Sabang _Cabayangan_*b*	Region IV-B	Samuro_siniloan	Region IV-A
Sibagat	Region XIII	Alman	Region III
		Pacol	Region IV-B
